# Palliative Radiation Therapy in the Treatment of Desmoplastic Small Round Cell Tumors

**DOI:** 10.7759/cureus.43863

**Published:** 2023-08-21

**Authors:** Jacfar Hassan, Aria Kieft, Michael Joiner, Steven Miller

**Affiliations:** 1 Radiation Oncology, Wayne State University School of Medicine, Detroit, USA

**Keywords:** whole abdominal radiation, systemic chemotherapy, pediatric cancers, desmoplastic small round blue cell tumours, palliative radiation therapy

## Abstract

An early adolescent male presented with six months of nausea, vomiting, and constipation. A chest computed tomography (CT) scan revealed multiple pulmonary nodules of varying sizes and a 3.1 cm pleural-based mass-like density in the right lower pulmonary lobe suspicious for metastatic disease. A CT scan of the abdomen and pelvis revealed diffuse metastatic disease involving the lungs, liver, and peritoneum. An ultrasound (US)-guided core needle biopsy of the liver was performed, and the morphology and immunohistochemistry were consistent with a poorly differentiated carcinoma. Further workup was performed, and the patient was diagnosed with a desmoplastic small round cell tumor (DSRCT). The patient underwent eight cycles of chemotherapy, but his tumor metastasized to distant sites. He then underwent two courses of palliative radiation therapy to the pelvis. His cancer continued to progress, and he eventually succumbed to his disease. This case report evaluates the evidence, data, radiation dosages, and techniques for palliative radiation therapy for DSRCTs.

## Introduction

Desmoplastic small round cell tumors (DSRCTs) are rare but highly aggressive sarcomas that predominantly affect adolescent and young adult males [1]. They are a part of the small round cell tumor family, which includes tumors, such as rhabdomyosarcoma, Wilms tumor, neuroblastoma, and Ewing sarcoma. They typically begin in the serosal surface of the abdomen and can metastasize to other parts of the body. DSRCTs are most commonly asymptomatic; however, they can present with abdominal pain and/or distention, constipation, or weight loss. We report a case of a DSRCT treated with chemotherapy and palliative radiation therapy.

## Case presentation

A previously healthy early adolescent male presented to our clinic complaining of severe epigastric and periumbilical abdominal pain, worsening fatigue, right sided chest pain, shortness of breath, and generalized body aches. He also had a decrease in stool output and caliber, and a 10-lb weight loss over two weeks. He had previously presented with complaints of six months of nausea, vomiting, and constipation at another hospital two weeks prior, where following investigation, he was diagnosed with stage IV metastatic colon cancer.

On examination, he was normotensive with a blood pressure of 129/91 and tachycardic with a heart rate of 111 bpm. Physical examination showed a distended abdomen with mild ascites. Positron emission tomography and computed tomography (PET/CT) revealed metastatic cancer with masses in the liver, peritoneum, and lung.

**Figure 1:**
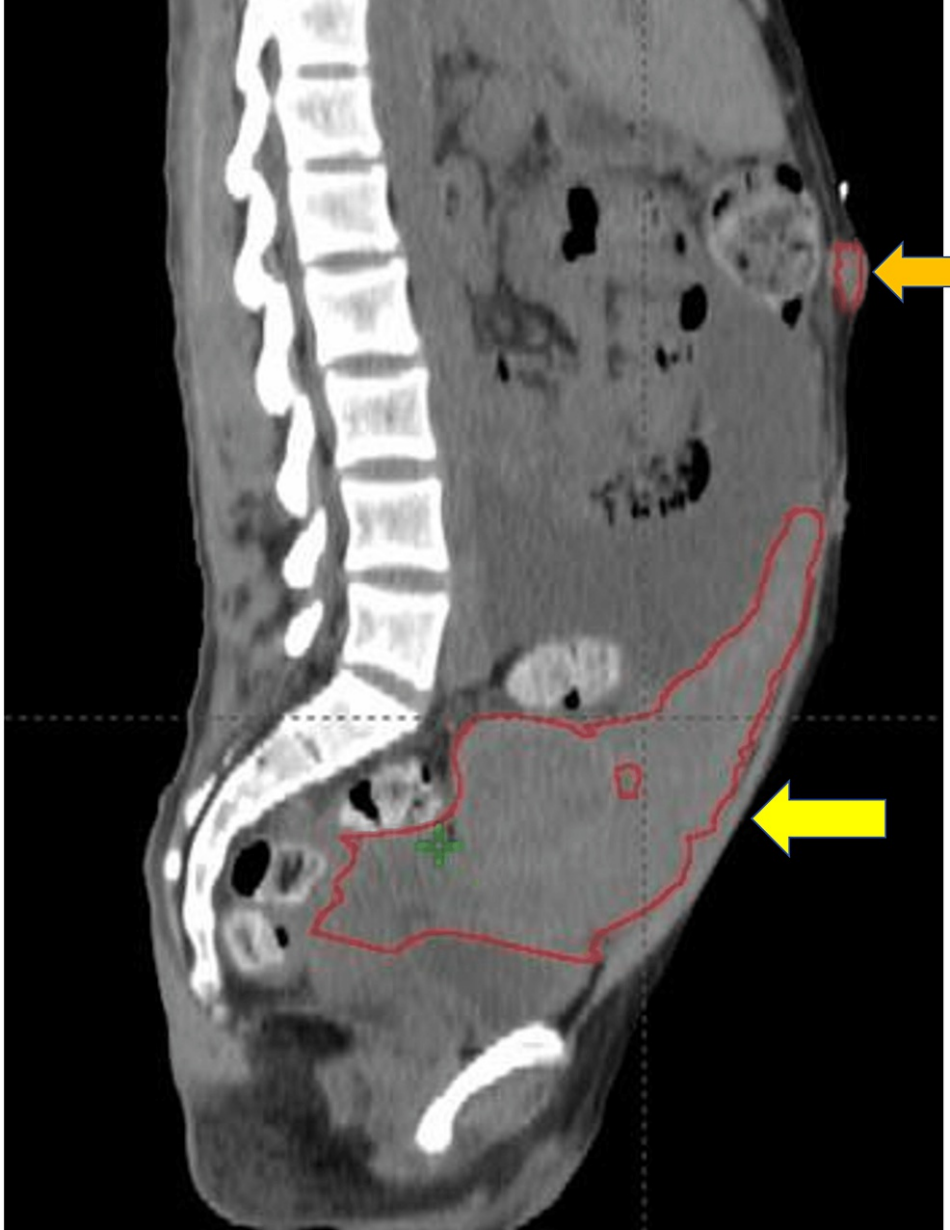
Sagittal CT with gross tumor volume (GTV) of subcutaneous nodule (orange arrow) and an abdominal and pelvic mass (yellow arrow).

Initial imaging studies and biopsy were performed at an outside hospital: Chest CT demonstrated multiple nodules of varying sizes and a 3.1 cm pleural-based, mass-like hyperdensity in the right lower pulmonary lobe suspicious for metastatic disease. There were enlarged right hilar and mediastinal lymph nodes measuring up to 1.8 cm. A CT of the abdomen and pelvis showed findings consistent with diffuse metastatic disease involving the lungs, liver, and peritoneum (Figure 1). A small amount of free fluid was also found within the pelvis and adjacent to the liver. Magnetic resonance imaging (MRI) of the brain and a testicular ultrasound were negative for abnormality.

An ultrasound (US)-guided core needle biopsy of the liver was performed. Multiple immunohistochemical stains were performed to characterize the nature of the lesion. The neoplastic cells were positive for pankeratin and special AT-rich sequence-binding protein 2 (SATB2) and negative for cytokeratin7 (CK7), cytokeratin 20 (CK20), CDX2, p40, synaptophysin, chromogranin, arginase, CD30, placental alkaline phosphatase (PLAP), and octamer-binding transcription factor (OCT4). While the origin of disease was uncertain, colorectal, upper gastrointestinal (GI), pancreaticobiliary, or lung origin were felt to be most likely. A recommendation for transfer to a tertiary care center was made.

**Figure 2:**
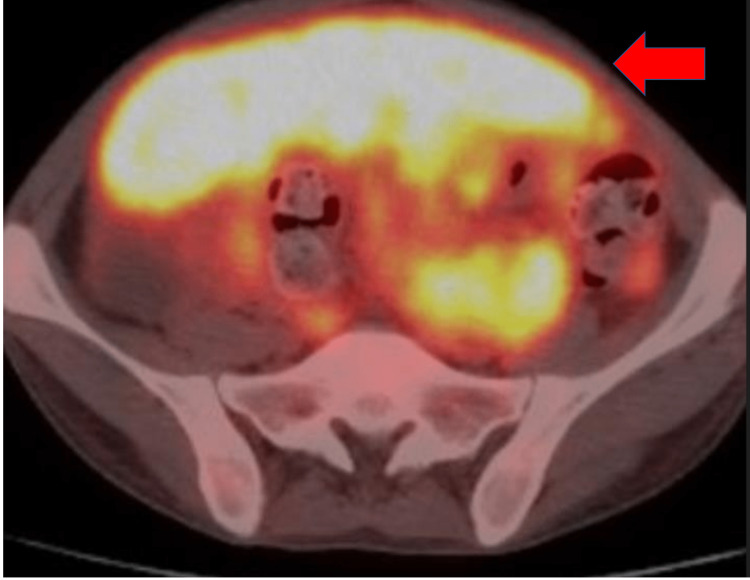
PET/CT demonstrating metastatic masses in the liver and abdomen along with mesenteric and peritoneal deposits (orange arrow) PET/CT: positron emission tomography and computed tomography

**Figure 3:**
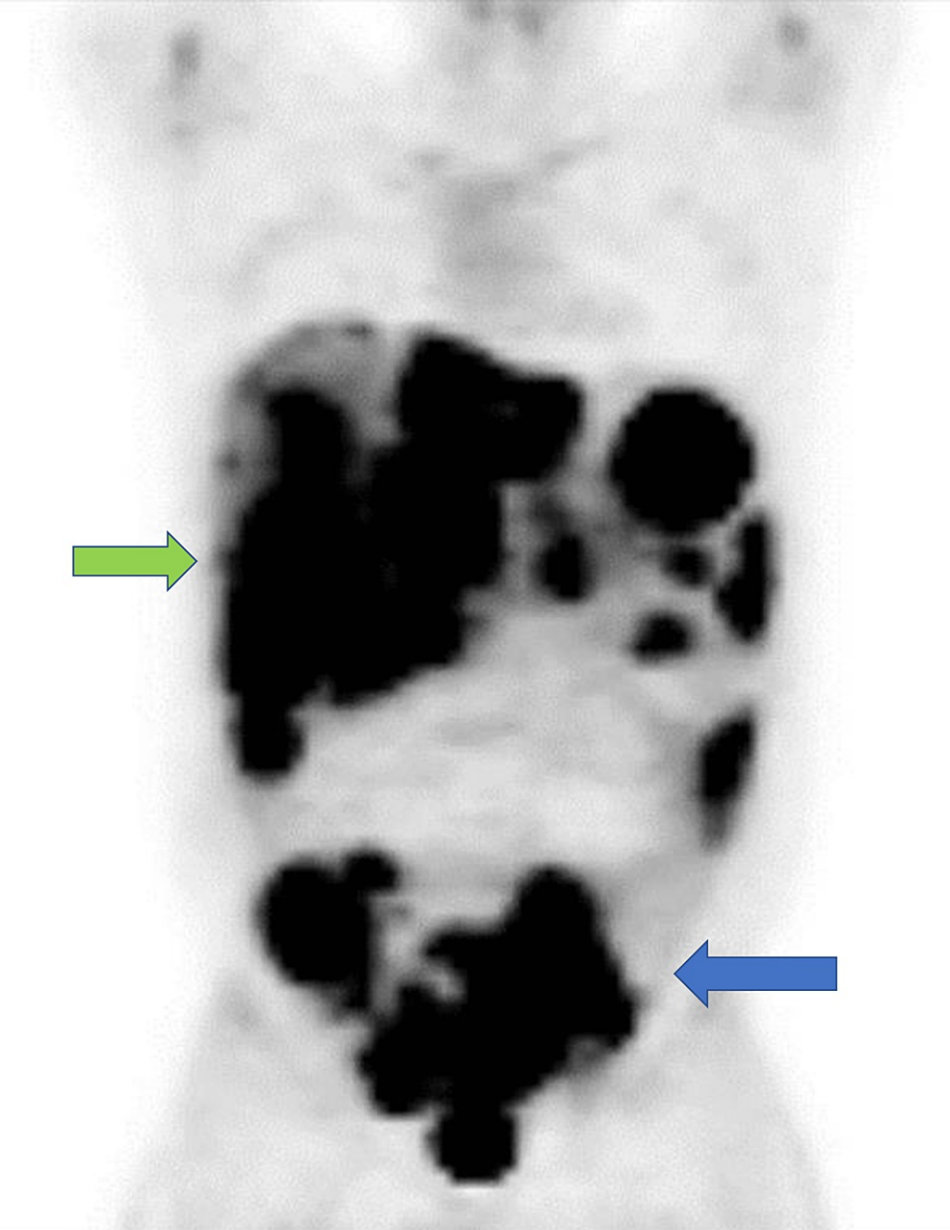
PET demonstrating metastatic masses in the liver and abdomen (green arrow) along with mesenteric and peritoneal deposits (blue arrow). PET: positron emission tomography

The patient was admitted to our facility two weeks later. He underwent esophagogastroduodenoscopy and colonoscopy with biopsy in continuation of diagnostic work-up. The colonoscopy revealed scattered whitish discolorations with friable mucosa of the rectum, but no evidence of primary colorectal carcinoma. A PET/CT scan was done, which revealed metastasis to the liver, mesenteric or peritoneal deposits, and masses in the abdomen, mediastinal, and hilar adenopathy, along with right pulmonary nodules and pleural deposits. The maximum standardized uptake value (SUVmax) was 9.9 and previously 5.6 for the lesion involving the right hepatic lobe and adjacent peritoneum (Figures 2, 3). 

**Figure 4:**
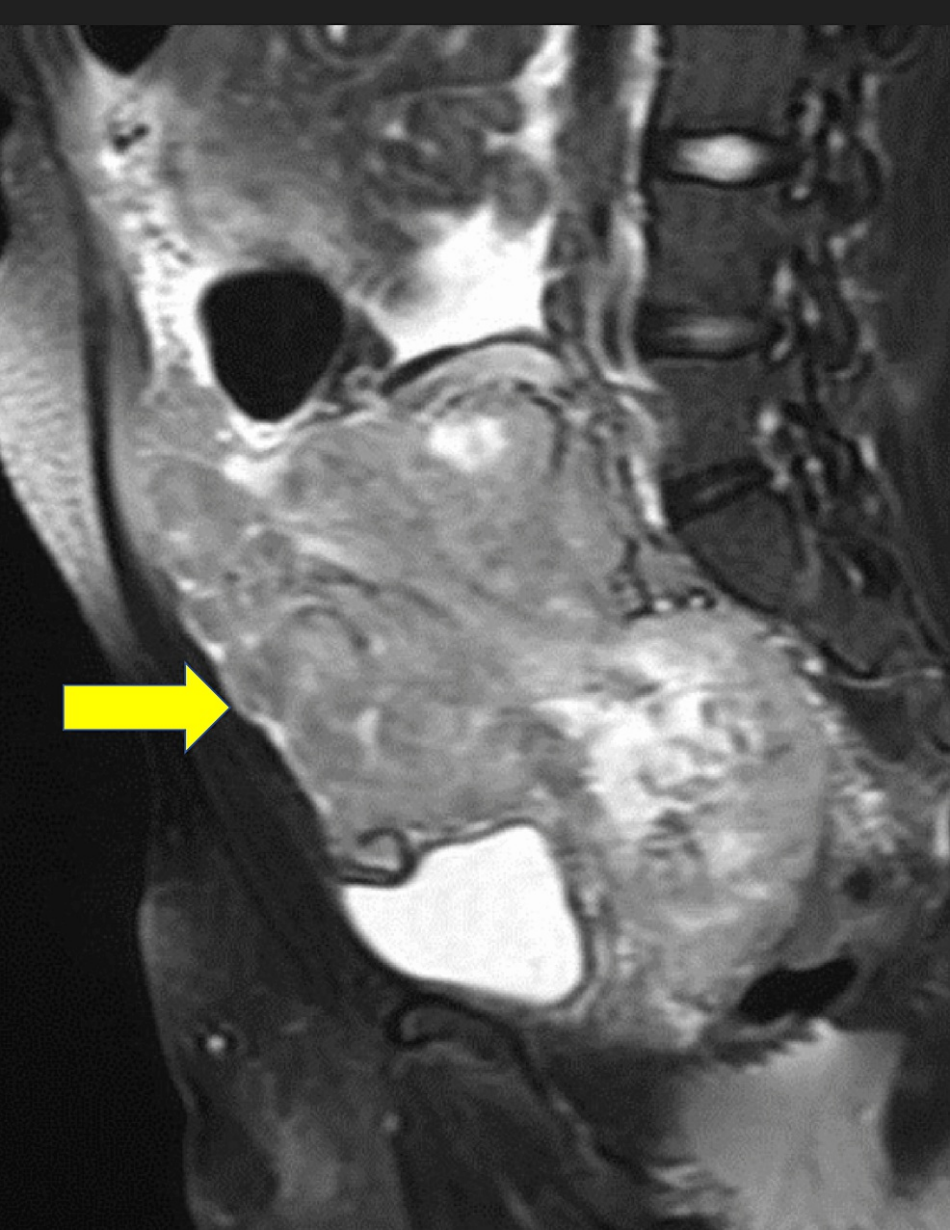
Sagittal T2 MRI demonstrating a pelvic mass (yellow arrow). MRI: magnetic resonance imaging

There was also a mass between the urinary bladder and rectum (Figure 4). This mass was thought to be the most likely site of the primary tumor, but a biopsy was not performed due to concerns for necrosis and the high risk of complications, such as seeding of the tumor. 

Repeated US-guided liver biopsy was completed and demonstrated an undifferentiated neoplasm. Specifically, the biopsy specimen consisted of hepatic parenchyma infiltrated by a proliferation of cells with scant cytoplasm and round to oval nuclei. Single-cell necrosis and larger areas of necrosis were also seen. The cells showed anti-CK (CAM 5.2) immunoreactivity, partial anti-CK monoclonal antibody (AE1/AE3) immunoreactivity, and dot-like immunoreactivity with vimentin. No immunoreactivity with cluster of differentiation 99 (CD99), desmin, neuron-specific enolase (NSE), glypican 3, hepar, Wilms tumor protein 1 (WT-1), or myogenin was observed. SMARCB1 (INI-1) immunoreactivity was retained. This indicated that the tumor was expressing mesenchymal and epithelial markers. These findings were consistent with desmoplastic round small cell tumor, which typically expresses epithelial, mesenchymal, and neural markers simultaneously. Tumor tissue was sent to Tempus, which confirmed the diagnosis of desmoplastic round cell tumor.

He received one cycle of palliative Ifosfamide, carboplatinum, and etoposide (ICE) chemotherapy prior to his diagnosis. He experienced Ifosfamide-associated central nervous system toxicity, which has been reported in 5% to 30% of patients treated with ifosfamide. Its pattern is characterized by metabolic encephalopathy with confusion, blurred vision, mutism, auditory or visual paranoid hallucinations, seizures, and rarely coma [2]. The ifosfamide central nervous system toxicity was treated with methylene blue at a dose of 50 mg every four hours. He was then switched to the Memorial Sloan Kettering Cancer Center P6 regimen (MSKCC P6) treatment protocol for desmoplastic round cell tumor for the next four cycles of his chemotherapy treatment. The MSKCC P6 protocol consists of five cycles of chemotherapy. Two days of high-dose cyclophosphamide and three days of one-hour infusion doxorubicin and vincristine (CDV) were given during cycles 1, 2, and 4. Cycles 3 and 5 consisted of ifosfamide and etoposide (IE) given as one-hour infusions on five consecutive days [3].

After the first cycle of MSKCC P6, the patient was hospitalized with febrile neutropenia along with worsening blood-streaked emesis and diarrhea. He also developed mucositis and thrombocytopenia. His condition improved with antibiotics and a platelet transplant. Upon completion of the second cycle of MSKCC P6, PET/CT and MRI showed decreased size and fluorodeoxyglucose (FDG) avidity of his disease. (An FDG avid area of superior right region of the peritoneum/liver boundary decreased in radiotracer uptake to an SUV 3.0 from 3.9 previously.) After his third cycle of MSKCC P6, he again developed febrile neutropenia and was treated with antibiotics and a platelet transplant. After his fourth cycle of MSKCC6, restaging PET/CT, CT abdomen/pelvis, and an MRI demonstrated a decrease in the size of the abdominal mass and metastatic disease and improvement in FDG avidity. He received temozolomide and irinotecan for his fifth and sixth cycles of chemotherapy. He received IE for his seventh and final cycle of MSK P6 regimen. He experienced Ifosfamide neurotoxicity again, so he was treated with methylene blue, and the dose was reduced.

CT abdomen and pelvis after the eighth cycle of chemotherapy showed new pulmonary nodules with redemonstration of abdominal masses with worsening ascites. There was also redemonstration of large loculated centrally hypodense pelvic masses with diffuse peritoneal deposits and hepatic metastatic disease. CT abdomen and pelvis also showed multiple areas of mildly prominent small and large bowel colonic wall thickening, which was slightly more pronounced at the region of the sigmoid colon, possibly consistent with colitis. He underwent a delayed nephogram, which demonstrated mild left hydronephrosis, likely secondary to obstruction and mass effect from the peritoneal metastases and ascites. There was also development of multiple new pulmonary nodules throughout the lungs, which were consistent with disease progression. Patient and family discussion included no relevant surgical options, but palliative chemotherapy, radiation, or both were offered. The oncology team, patient, and his family agreed to discontinue MSK P6 protocol because of likelihood of prolonged immunosuppression and possibility of redeveloping febrile neutropenia and severe mucositis.

**Figure 5:**
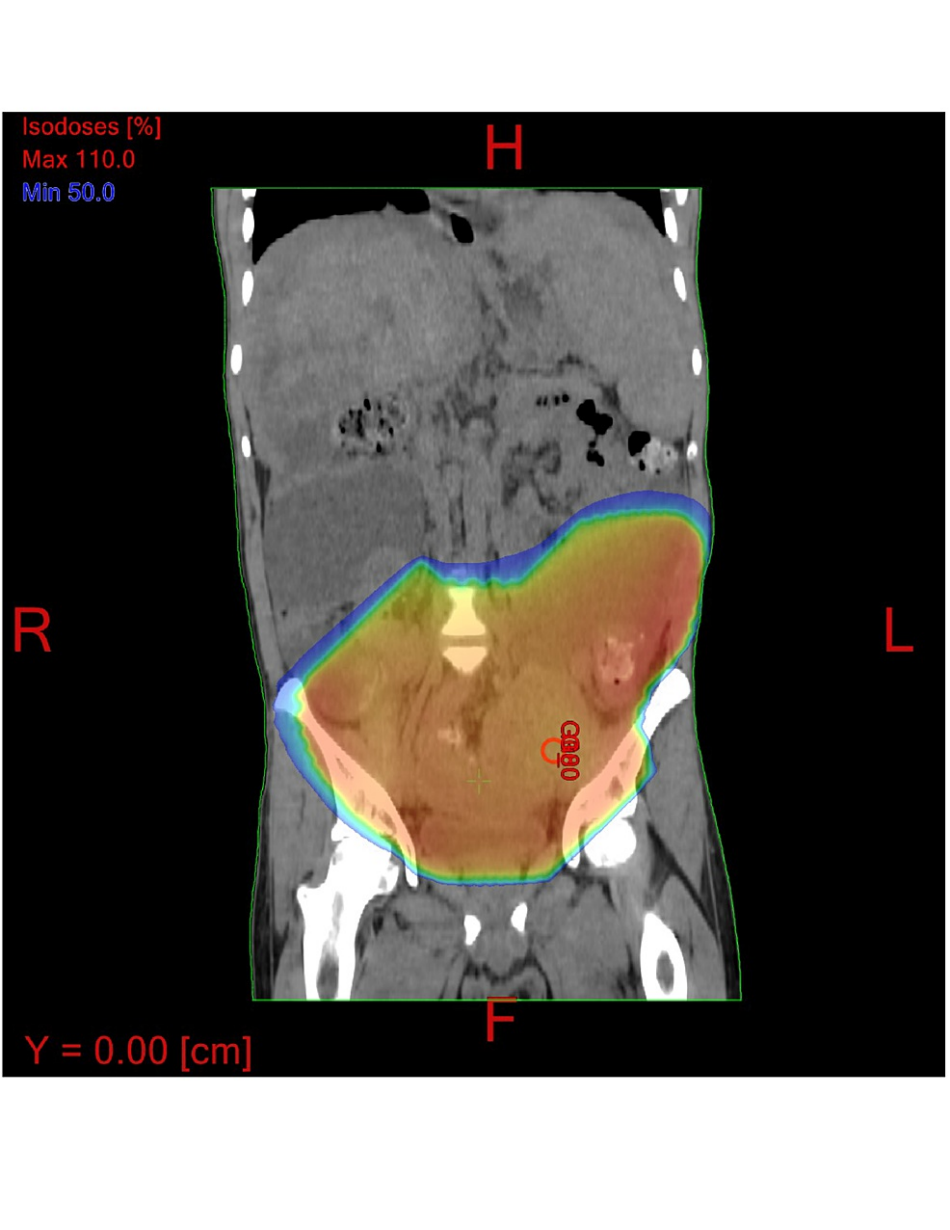
Radiation treatment fields with the orange area representing the treatment volume.

The patient was endorsing worsening pain in the lower abdomen/pelvis, with associated urinary/bowel straining. These symptoms corresponded with PET/CT imaging that demonstrated an enlarging pelvic mass anterior to the rectum that was believed to be the primary tumor. He also had a painful skin nodule on his abdomen. A course of radiotherapy targeting the pelvic mass and skin nodule was recommended for palliation. The patient agreed to proceed with palliative radiotherapy concurrent with oral chemotherapy and targeted therapy with a tyrosine kinase inhibitor (cabozantinib). He underwent 800 cGy in a single fraction to the pelvis using a two-field technique consisting of anterior-posterior/posterior-anterior (AP/PA) fields and an electron patch to treat the skin nodule. He was then given cabozantinib and initiated a second round of palliative radiation therapy approximately nine months later to the abdominal mass. He was scheduled to undergo a course of palliative radiation therapy to a total dose of 2000 cGy in five fractions (Figure 5).

However, he only underwent three out of the five planned fractions secondary to worsening of his condition and entering hospice. A week after the discontinuation of palliative radiation therapy, the patient developed severe hyponatremia. His cancer also continued progressing despite chemotherapy and radiation. The patient was advised that his level of hyponatremia could result in seizures and would have to be corrected slowly in the intensive care unit (ICU). The palliative care team discussed options with the patient and his family. It was ultimately decided that intervention in the ICU would not be in his best interest and might cause more suffering, so he was discharged home. The patient expired a month later at home with hospice support.

## Discussion

DSRCTs are rare, aggressive soft tissue sarcomas of mesenchymal origin that often present with multiple intrabdominal tumors [4]. They also exhibit a multi-phenotypic pattern of immunohistochemical staining. This disease predominantly originates from the peritoneum or retroperitoneum and can invade the omentum. They often present with multiple peritoneal implants involving the diaphragm, splenic hilum, mesentery of the small and large bowel, and pelvic peritoneum [5]. Patients with abdominal tumors are often diagnosed with advanced disease as most are asymptomatic until progression to bulky metastatic disease with extensive seeing in the visceral and parietal peritoneum. Advanced disease is associated with symptoms of pain, ascites, constipation, weight loss, distention, and jaundice. Approximately half of the patients diagnosed with DSRCTs will have extraperitoneal metastases at the time of presentation [1].

Patients with abdominal tumors are often diagnosed with advanced disease as most are asymptomatic until progression to bulky metastatic disease with extensive seeing in the visceral and parietal peritoneum. Advanced disease is associated with symptoms of pain, ascites, constipation, weight loss, distention, and jaundice. Approximately half of patients diagnosed with DSRCTs will present with extraperitoneal metastases at the time of presentation.

The prognosis for this disease is poor with the five-year overall survival estimated at only 15-30%. Approximately 60-70% of patients die secondary to disease progression within three years of diagnosis [1]. The current treatment regimen for DSRCTs is chemotherapy, resection, and radiation therapy [4]. In a study of 250 patients with DSRCTs, it was found that treatment with stem cell transplant in remission, complete surgical resection, and radiation had significantly improved overall survival at three to five years [6]. A second study of 66 patients with DSRCTs showed that chemotherapy, surgery, and radiation therapy together produced a 55% overall survival at three years compared to 27% if one of these therapeutic modalities was missing [5].

In one case of a patient with a DSRCT, the patient was a 38-year-old male that presented with multiple solid cystic mass lesions in the peritoneal cavity and pelvis and a small amount of ascites [6]. There was also a confluent solid mass located in the upper and lower abdomen, which demonstrated moderate heterogenous enhancement. Several 20 x 20 mm nodules were also found in the retroperitoneal region and the peritoneal and pelvic cavities, which was suggestive of multiple metastases. The patient was treated with radiation therapy with a total dosage of 40 Gy in 20 fractions. The patient was also put on concurrent CAP multiagent systemic chemotherapy consisting of cyclophosphamide, adriamycin, and cisplatin, which was administered for three cycles. Following supportive care, the patient’s symptoms rapidly alleviated. The patient eventually succumbed to significant complications induced by recurrence and metastases 30 months after treatment.

In another case of a patient with a DSRCT, the patient was an early adolescent male that presented with upper abdominal pain [7]. He had a palpable mass measuring approximately 4 cm in diameter in the middle of the lower abdominal region. Abdominal CT showed the presence of a lower abdominal mass measuring 4 cm, hepatomegaly, and multiple intrahepatic metastasis. Biopsy of the intrahepatic masses showed proliferation of atypical cells with hyperchromatic nuclei forming irregularly shaped small nests accompanied by fibromyxoid stroma. The characteristic reciprocal translation specific to DSRCTs was identified on fluorescence in situ hybridization (FISH), so a diagnosis of DSRCT was made. The patient was treated with six rounds of chemotherapy consisting of carboplatin and paclitaxel. His general condition recovered and was sustained for two months before his tumor enlarged six months later. He was then treated with a second-line chemotherapy P6 protocol consisting of seven courses of chemotherapy with cyclophosphamide, doxorubicin, and vincristine. He responded transiently to the chemotherapy before his condition deteriorated and he died approximately 20 months after receiving his initial diagnosis.

The treatment paradigm used for DSRCTs should be decided based on the progression and location of the patient's cancer and the patient’s wishes. Surgery was not an option for our patient because of the diffuse nature of his disease. The patient initially responded to chemotherapy with a decrease in the size of the primary tumor and metastatic disease. Unfortunately, the patient's cancer continued progressing despite the chemotherapy. The radiation therapy used in this case was palliative and without apparent benefit. This may have been due to the very late stage of the disease process at which time he was referred or to the treatment resistance of his disease.

## Conclusions

DSRCTs are rare but highly aggressive sarcomas. Most patients are diagnosed in an advanced stage with large masses and/or extensive seeding in the visceral and parietal peritoneum. The current treatment paradigm used for DSRCTs is surgery, chemotherapy, and radiation. The treatment plan for patients should be decided on a case-to-case basis depending on the progression and location of the patient's cancer and the patient’s wishes.
